# Misfolded N-CoR is Linked to the Ectopic Reactivation of CD34/Flt3-Based Stem-Cell Phenotype in Promyelocytic and Monocytic Acute Myeloid Leukemia

**DOI:** 10.3389/fonc.2015.00210

**Published:** 2015-10-07

**Authors:** Dawn Sijin Nin, Feng Li, Sridevi Visvanathan, Matiullah Khan

**Affiliations:** ^1^Department of Biochemistry, Yong Loo Lin School of Medicine, National University of Singapore, Singapore, Singapore; ^2^Department of Pharmacology, Yong Loo Lin School of Medicine, National University of Singapore, Singapore, Singapore; ^3^Department of Biochemistry, School of Medicine, AIMST University, Semeling, Malaysia; ^4^Department of Pathology, School of Medicine, AIMST University, Semeling, Malaysia

**Keywords:** AML, NCoR, misfolded protein, Flt3, CD34, LSCs

## Abstract

Nuclear receptor co-repressor (N-CoR) is the key component of generic co-repressor complex essential for the transcriptional control of genes involved in cellular hemostasis. We have recently reported that N-CoR actively represses *Flt3*, a key factor of hematopoietic stem cells (HSC) self-renewal and growth, and that de-repression of *Flt3* by the misfolded N-CoR plays an important role in the pathogenesis of promyelocytic and monocytic acute myeloid leukemia (AML). The leukemic cells derived from the promyelocytic and monocytic AML are distinctly characterized by the ectopic reactivation of stem cell phenotypes in relatively committed myeloid compartment. However, the molecular mechanism underlying this phenomenon is not known. Here, we report that N-CoR function is essential for the commitment of primitive hematopoietic cells to the cells of myeloid lineage and that loss of N-CoR function due to misfolding is linked to the ectopic reactivation of generic stem cell phenotypes in promyelocytic and monocytic AML. Analysis of *N-CoR* and *Flt3* transcripts in mouse hematopoietic cells revealed a positive correlation between *N-CoR* level and the commitment of myeloid cells and an inverse correlation between *N-CoR* and *Flt3* levels in primitive as well as committed myeloid cells. Enforced N-CoR expression in mouse HSCs inhibited their growth and self-renewal potentials and promoted maturation toward cells of myeloid lineage, suggesting a role of N-CoR in the commitment of cells of myeloid lineage. In contrast to AML cells with natively folded N-CoR, primary and secondary promyelocytic and monocytic AML cells harboring the misfolded N-CoR were highly positive for Flt3 and myeloid antigen-based HSC marker CD34. Genetic and therapeutic restoration of N-CoR conformation significantly down-regulated the CD34 levels in monocytic AML cells, suggesting an important role of N-CoR in the suppression of CD34-based HSC phenotypes. These findings collectively suggest that N-CoR is crucial for the commitment of primitive hematopoietic cells to cells of myeloid lineage and that misfolded N-CoR may contribute to transformation of committed myeloid cells through the ectopic reactivation of Flt3/CD34-based stem cell phenotypes in promyelocytic and monocytic AML. Moreover, these findings provide novel mechanistic insights into the formation of leukemic stem cells in subsets of AML and identify the misfolded N-CoR as a subtype-specific biomarker of AML.

## Introduction

Balanced transcriptional control of self-renewal and lineage-specific genes by the coordinated actions of co-activator and co-repressor proteins and sequence-specific transcriptional factors plays an important role in the normal growth and maturation of hematopoietic cells. During the highly regulated process of commitment of primitive myeloid cells in the hematopoietic system, the co-activator proteins confer the specific lineage identity by triggering the expression of genes essential for lineage commitment, whereas the co-repressor proteins concurrently suppress the cellular self-renewal potentials by actively repressing the self-renewal genes (1). Thus, the maturation and commitment of primitive hematopoietic cells is achieved through the coordinated actions of these co-activator and co-repressor proteins, and any imbalance in this coordination could alter the linear growth and maturation of hematopoietic cells, ultimately leading to malignant growth and transformation in AML (acute myeloid leukemia). One of the key components of the generic co-repressor protein complex essential for the transcriptional repression of various growth-promoting genes, including the self-renewal genes, is nuclear receptor co-repressor (N-CoR), which was first identified as a co-repressor of unliganded nuclear hormone receptors (2, 3). We have previously shown that N-CoR is a direct target of heterogeneous oncogenic insults such as fusion oncogenes created by chromosomal translocations, which trigger a nearly homogenous change in N-CoR conformation through aberrant post-translational modification, ultimately leading to its misfolding and premature loss in promyelocytic and monocytic AML, two distinct subtypes of AML characterized by maturation arrest of intermediate myeloid cells (4–9). As shown by us, the N-CoR misfolding in monocytic AML-derived primary and secondary leukemic cells was actually trigged by Akt-induced phosphorylation of N-CoR at the consensus Akt motif located in the c-terminal domain of N-CoR protein (9). We have also demonstrated that Flt3, the cytokine receptor essential for the self-renewal of primitive hematopoietic cells (10, 11), is actively repressed by N-CoR and that misfolding and premature loss of N-CoR protein leads to the de-repression of Flt3 in promyelocytic and monocytic AML-derived primary and secondary leukemic cells (8). These findings suggested that N-CoR-mediated transcriptional repression of stem cell genes like *Flt3* might be crucial for the suppression of self-renewal potential of hematopoietic cells during their commitment and differentiation to cells of myeloid lineage and that de-repression of *Flt3* due to N-CoR misfolding may contribute to formation of leukemia-initiating cells (LICs) or leukemic stem cells (LSCs) through the ectopic reactivation of self-renewal potentials in relatively matured cells.

Although AML is increasingly being recognized as a stem cell disorder, the true origin of LSCs in AML is still a matter of debate. It is not clear whether LSCs in AML are initiated in the primitive hematopoietic stem cell compartment or they merely represent a re-acquisition of stem cell-like characteristics in relatively committed myeloid cells. Several studies in mice have suggested that LICs in promyelocytic AML could arise in the committed progenitor cells (12–15). Likewise, it has recently been shown that some monocytic AML-specific chromosomal translocations impart stem cell-like properties only on the committed progenitor cells and that LSCs in monocytic AML are initiated in the matured myeloid cell compartment when these matured cells ectopically regain the stem cell-like properties (16, 17). However, how these so-called stem cell-like properties are kept in check when the primitive hematopoietic cells progress toward commitment and maturation and how exactly these properties are temporally reactivated or unmasked in promyelocytic and monocytic AML are not known. One of the important and most basic phenotypes based on which both the normal hematopoietic stem cells and LSCs in various AML subtypes are characterized is the cell surface expression of myeloid antigen-based stem cell marker CD34. As with the activity of hematopoietic stem cells, the LSC activity in some specific subtypes of AML are also contained within the CD34^+^ fraction of AML cells (18–22), making it a fundamental stem cell marker for both HSCs and LSCs. However, leukemic cells derived from various AML subtypes display significant heterogeneity based on CD34 level. Here, we report that transcriptional repression mediated by N-CoR is essential for the suppression of growth and self-renewal potentials of HSCs and that loss of N-CoR function due to misfolding leads to ectopic reactivation of Flt3 and CD34-based hematopoietic stem cell phenotypes in promyelocytic and monocytic AML. These findings suggest that transcriptional repression mediated by N-CoR might be crucial for the suppression of self-renewal potentials of primitive hematopoietic cells during their commitment and maturation to cells of myeloid lineage, and abrogation of this repression due to the misfolding and premature loss of N-CoR may contribute to the formation of LSC or LIC through the ectopic reactivation of CD34^+^/Flt3^+^-based stem cell phenotype in promyelocytic and monocytic AML.

## Results

### N-CoR inhibits the self-renewal potential of primitive hematopoietic cells

**Figure 1:**
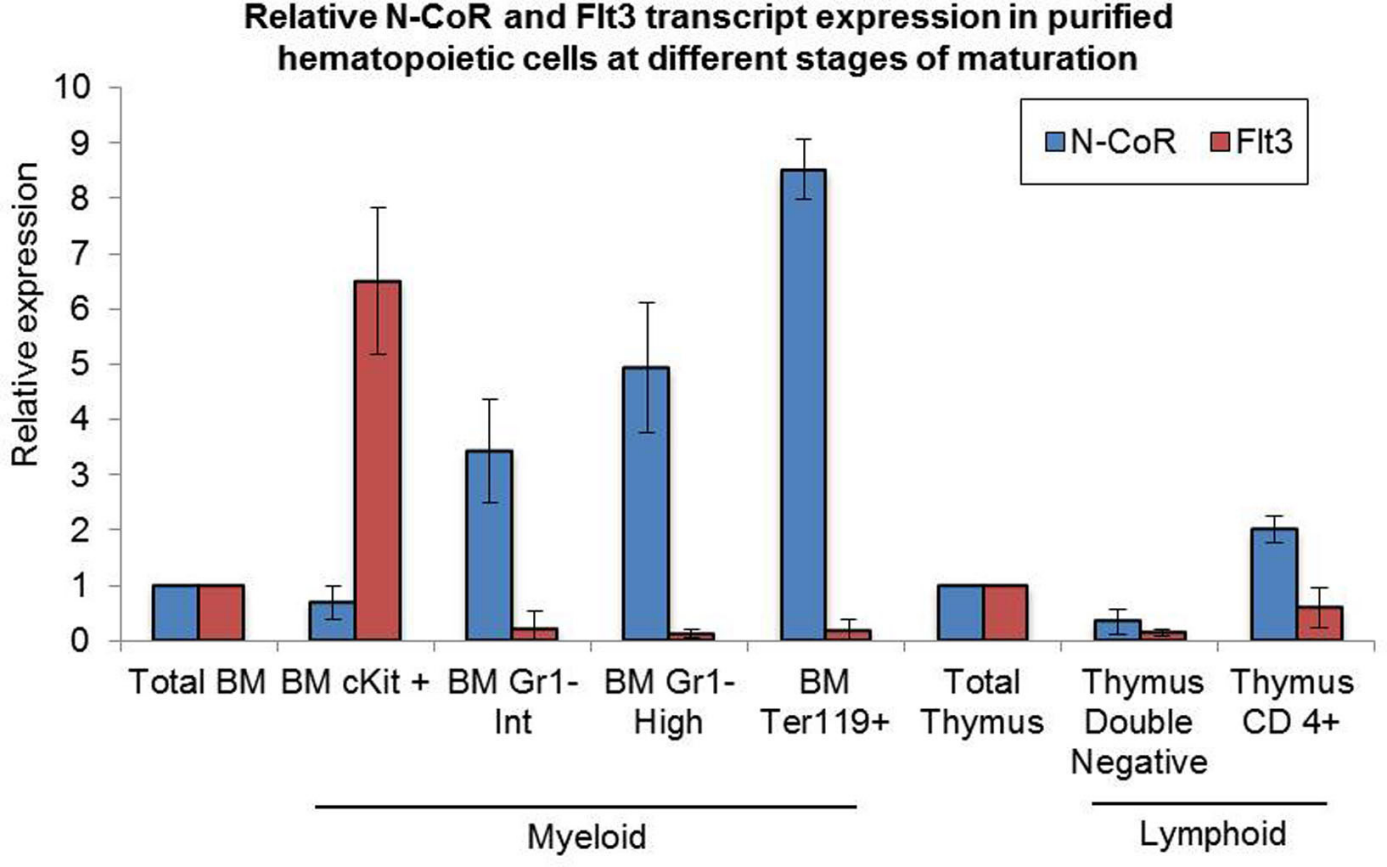
**Levels of N-CoR and Flt3 transcripts in purified mouse hematopoietic cells**. Levels of N-CoR and Flt3 transcripts in total bone marrow as well as in each purified subtypes of mouse hematopoietic cells were determined by real-time RT-PCR analysis. The values presented in the graph are average of three independent experiments.

**Figure 2:**
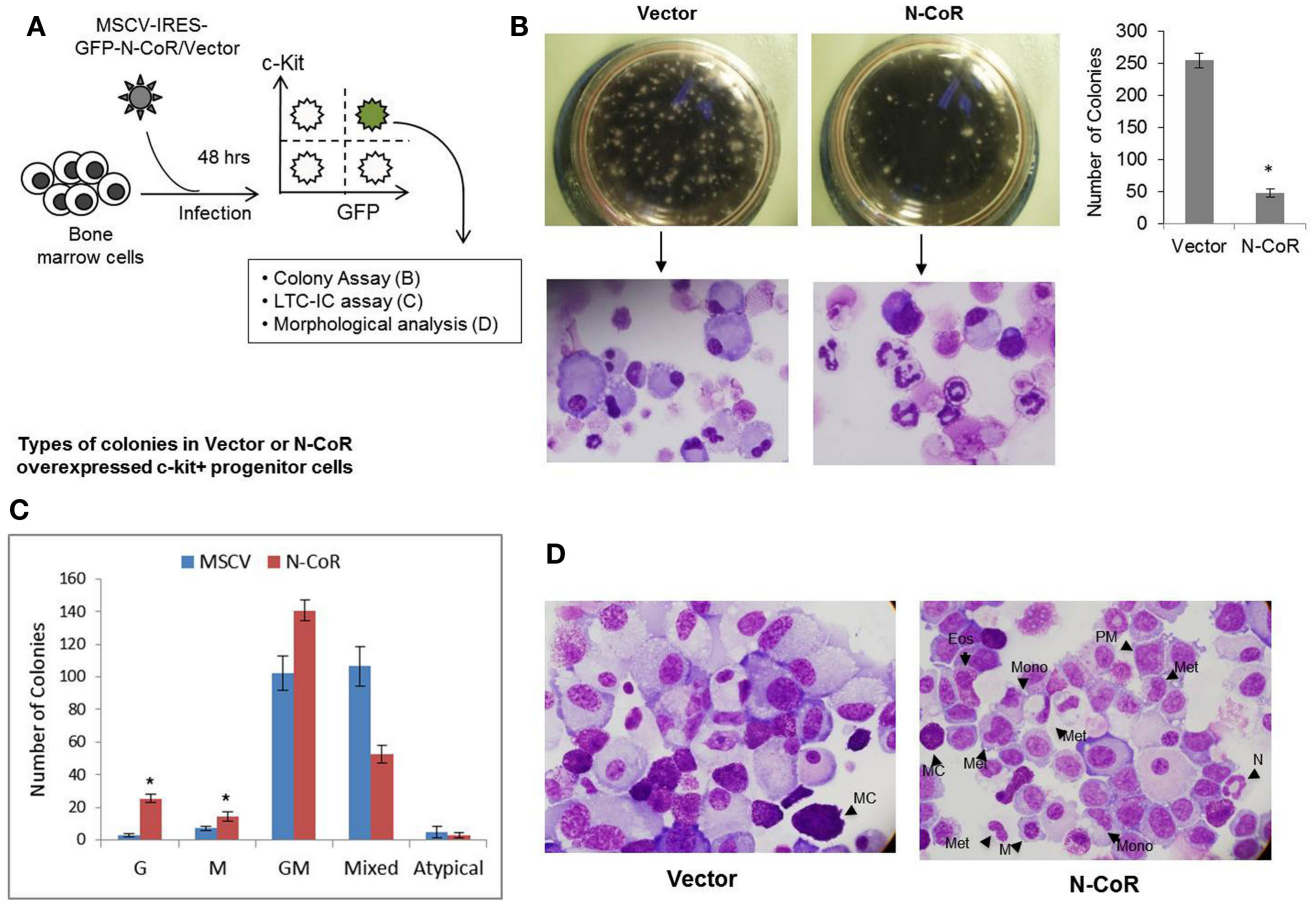
**Enforced N-CoR expression in c-Kit^+^ stem cell/progenitor cells inhibits their growth and promotes commitment toward myeloid lineage**. **(A)** Schematic representation of the experiments performed with mouse bone marrow cells transduced with N-CoR or control vector. **(B)** Enforced N-CoR expression in c-Kit^+^ stem cell/progenitor cells inhibits their self-renewal potential. The growth and commitment of mouse primitive hematopoietic cells transduced with MSCV-IRES-GFP-N-CoR or empty vector were determined in colony formation assay and Wright–Giemsa staining. The number of colonies derived from vector or N-CoR-infected cells (left panel) was counted and data were plotted as bar graph (right panel). Asterisk represents statistical significance in Student’s *t*-test, *p* < 0.01. Error bars indicate mean ± SD. **(C)** Colony-forming unit (CFU)-culture assay: the number of granulocyte (G), macrophage (M), granulocyte–macrophage (GM), and mixed colonies (granulocyte, erythroid, monocyte, and megakaryocyte) formed by GFP- and c-Kit-positive cells treated with G-CSF in methylcellulose medium for 10 days was scored and plotted as bar graph. Asterisks represent statistical significance in Student’s *t*-test, *p* < 0.01. Error bars indicate mean ± SD. **(D)** Morphology of purified mouse bone marrow cells transduced with MSCV-IRES-GFP-N-CoR or control vector was determined with Wright–Geimsa staining where eosinophils (Eos), mast cells (MC), meta-myelocytes (Met), monocytes (Mono), myelocytes (M), neutrophils (N), and promyelocytes (PM) are labeled (right panel).

We have recently shown that natively folded N-CoR actively represses the *Flt3* gene and that misfolded conformation-dependent loss of N-CoR leads to up-regulation of Flt3 in leukemic cells derived from promyelocytic and monocytic AML (8). To understand the significance of N-CoR-induced transcriptional repression of *Flt3* in the normal growth and maturation of primitive hematopoietic cells, we first determined the levels of N-CoR and Flt3 transcripts in mature and immature hematopoietic cells isolated from mouse bone marrow. Using multiparameter flow cytometry, these hematopoietic cells at various stages of development were isolated based on the expression of lineage-specific cell surface markers, and the level of N-CoR and Flt3 transcripts in each isolated cell type was determined by real-time PCR analysis. As shown in Figure 1, the level of Flt3 transcript in c-kit^+^ immature hematopoietic cells was significantly higher when compared with the level of N-CoR transcript. By contrast, the level of N-CoR transcript was significantly higher in relatively matured hematopoietic cells such as monocytes, granulocytes, and erythrocytes, in which the level of Flt3 transcript is significantly low (Figure 1). These findings suggested an inverse correlation between N-CoR and Flt3 expression during the growth and maturation of mouse hematopoietic cells. The gradual increase in N-CoR transcript level as the primitive hematopoietic cells progressed to maturation toward cells of myeloid lineage suggested a possible link between N-CoR expression and the maturation of primitive hematopoietic cells to cells of myeloid lineage. The inverse correlation in N-CoR and Flt3 levels observed in myeloid compartment was not quite evident in lymphoid compartment such as double-negative or CD4-positive lymphocytes derived from thymus (Figure 1). To further explore the role of N-CoR in the commitment of cells of myeloid lineage, the functional effect of ectopic N-CoR on the growth and maturation of c-kit^+^ hematopoietic cells, in which N-CoR was barely detected, was determined. First, mouse bone marrow cells were infected with retrovirus-based N-CoR expression plasmid MSCV-IRES-GFP-N-CoR or control empty vector MSCV-IRES-GFP. Next, virus-infected c-kit^+^ bone marrow cells were purified using c-kit^+^ antibody and GFP signal employing fluorescence-activated cell sorting (FACS). Finally, the growth and maturation of FACS-purified c-kit^+^ N-CoR-infected cells were compared with that of c-kit^+^ vector-infected cells through various functional assays as outlined in Figure 2A. First, to assess whether enforced N-CoR expression in the primitive hematopoietic cells could inhibit their growth and proliferation, 1 × 10^4^ FACS-purified c-Kit^+^ and GFP-positive cells were plated on methylcellulose medium and their growth was monitored by counting the number of colonies that grew in size due to the proliferation of cells. After 10 days of culture on methylcellulose medium, the MSCV-IRES-GFP-N-CoR-infected c-Kit^+^ cells made significantly fewer colonies when compared with cells infected with empty vector MSCV-IRES-GFP, suggesting that ectopic N-CoR might have suppressed the growth and proliferation of these cells (Figure 2B, upper panels). More significantly, morphological analysis of cells isolated from N-CoR- or vector-infected colonies revealed that N-CoR-infected colonies were highly enriched with matured myeloid cells when compared with colonies of vector-infected cells, suggesting a role of ectopic N-CoR in the maturation of hematopoietic cells to myeloid lineage (Figure 2B, lower panels). Next, to test whether enforced N-CoR expression in primitive hematopoietic cells could inhibit their self-renewal potentials at the expense of lineage commitment, the morphology of MSCV-IRES-GFP-N-CoR-infected c-Kit^+^ cells cultivated on OP9 stroma in the presence of GSCF, IL3, SCF, and EPo was analyzed through long-term culture-initiating cell (LTC-IC) assay. The LTC-IC assay is a well-established *in vitro* assay that measures the self-renewal and differentiation potentials of the primitive hematopoietic cells (23). LTC-ICs present in purified cell suspensions and co-cultured on a supportive feeder layer are detected by their sustained ability to produce hematopoietic progenitors (colony-forming cells) after around 4 weeks of culture. In the LTC-IC assay performed with FACS-purified MSCV-IRES-GFP-N-CoR or MSCV-IRES-GFP cells, the MSCV-IRES-GFP-N-CoR-infected cells generated significantly higher number of granulocyte (G) and macrophage (M) colonies when compared with cells infected with empty vector MSCV-IRES-GFP (Figure 2C). While MSCV-IRES-GFP-N-CoR-infected cells also generated slightly higher number of granulocyte–macrophage (GM) colonies, they produced significantly lower number of mixed or GEMM (granulocyte/erythroid/monocyte/megakaryocyte) colonies which are enriched in multipotent progenitor cells. These findings suggest that N-CoR may exert its role downstream of GEMM development. Next, we compared the morphology of MSCV-IRES-GFP-N-CoR-infected cells of LTC-IC assay with the cells infected with empty vector. As shown in Figure 2D, significantly higher number of myeloid lineage cells were present in MSCV-IRES-GFP-N-CoR-infected colonies when compared with colonies infected with empty vector (Figure 2D). Together, these findings suggested that enforced N-CoR expression in c-Kit^+^ primitive hematopoietic cells significantly reduced their self-renewal potentials and promoted their maturation to cells of myeloid lineage.

### N-CoR inhibits the repopulating potential of primitive hematopoietic cells

**Figure 3:**
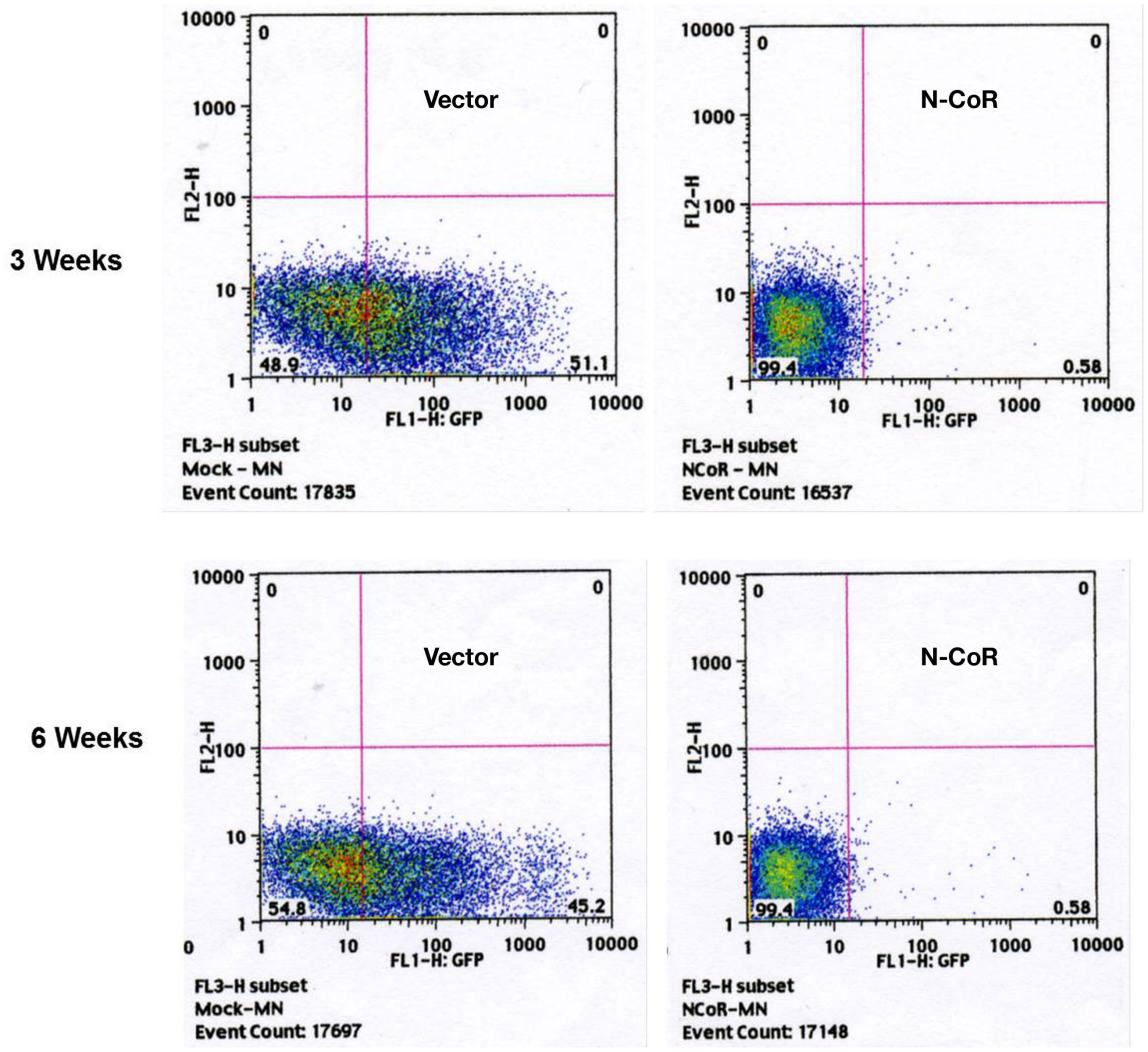
**Ectopically expressed N-CoR inhibits the growth and repopulating potential of c-Kit^+^ stem cell/progenitor cells *in vivo***. 5 × 10^5^ N-CoR or control vector-transfected BM cells were transplanted intravenously into sublethally irradiated C57BL/6 mice (8 Gy). The percentage of GFP-positive cells in the peripheral blood of transplanted mice was determined by FACS at the intervals of weeks 3 (upper panel) and 6 (lower panel).

One of the important functional properties based on which hematopoietic stem cells are characterized is their ability to repopulate the host hematopoietic system after transplantation in immunocompromised mice. Therefore, to determine if N-CoR could inhibit the ability of c-kit^+^ primitive hematopoietic cells to repopulate the hematopoietic system *in vivo*, a bone marrow transplantation assay was performed in immunosuppressed mice. 5 × 10^5^ FACS-purified MSCV-IRES-GFP-N-CoR or vector-infected cells were implanted intravenously into sublethally irradiated recipient mice and the number of GFP-positive cells in the peripheral blood of the recipient mice was analyzed by FACS at the interval of 3, 6, and 9 weeks. From 3 weeks onward, the number of GFP-positive cells in the peripheral blood of N-CoR transplanted mice was significantly lower when compared with mice transplanted with control vector (Figure 3), suggesting that enforced N-CoR expression in the primitive hematopoietic cells significantly reduced their ability to repopulate the hematopoietic system. These findings suggested that N-CoR could play an important role in the suppression of self-renewal potentials of primitive hematopoietic cells *in vivo*.

### Misfolded N-CoR is linked to the reactivation of CD34^+^/Flt3^+^-based stem cell phenotype in promyelocytic and monocytic AML

**Figure 4:**
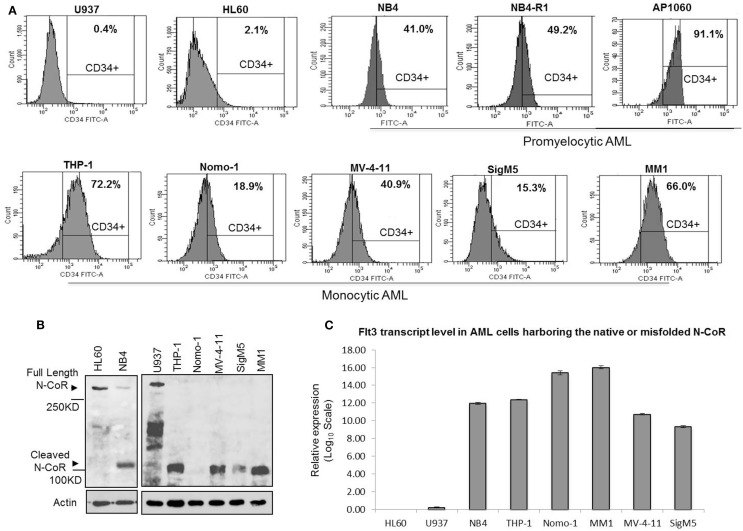
**Misfolded N-CoR is correlated to the CD34^+^/Flt3^+^-based stem cell phenotype in promyelocytic and monocytic AML-derived cells**. **(A)** Percentage of CD34^+^ cells in promyelocytic AML (NB4, NB4-R1, and AP1060) or monocytic AML (THP-1, Nomo-1, MV-4-11, SigM5, and MM1) as well as non-promyelocytic/monocytic AML cells (HL-60 and U937) as determined by flow cytometry. **(B)** An aliquot of whole-cell extract prepared from various AML-derived cells as mentioned on the top of each lane was resolved in SDS-PAGE and stained with anti-N-CoR antibody. All the CD34^+^ promyelocytic and monocytic AML cells (NB4, THP-1, Nomo-1, MV-4-11, SigM5, and MM1) harbor a distinctly misfolded N-CoR protein as demonstrated by its premature loss in Western blotting. In contrast, N-CoR in CD34-negative non-promyelocytic/monocytic AML cells (HL60 and U937) is natively folded and stable. For loading control, an aliquot of whole-cell extract was stained with residual protein beta-actin. **(C)** Level of Flt3 transcript in promyelocytic and monocytic AML (NB4, THP-1, Nomo-1, MV-4-11, SigM5, and MM1) and non-promyelocytic/monocytic AML cells (HL60 and U937) was determined by real-time quantitative PCR and plotted as bar graph. The values presented are average of three independent experiments.

**Figure 5:**
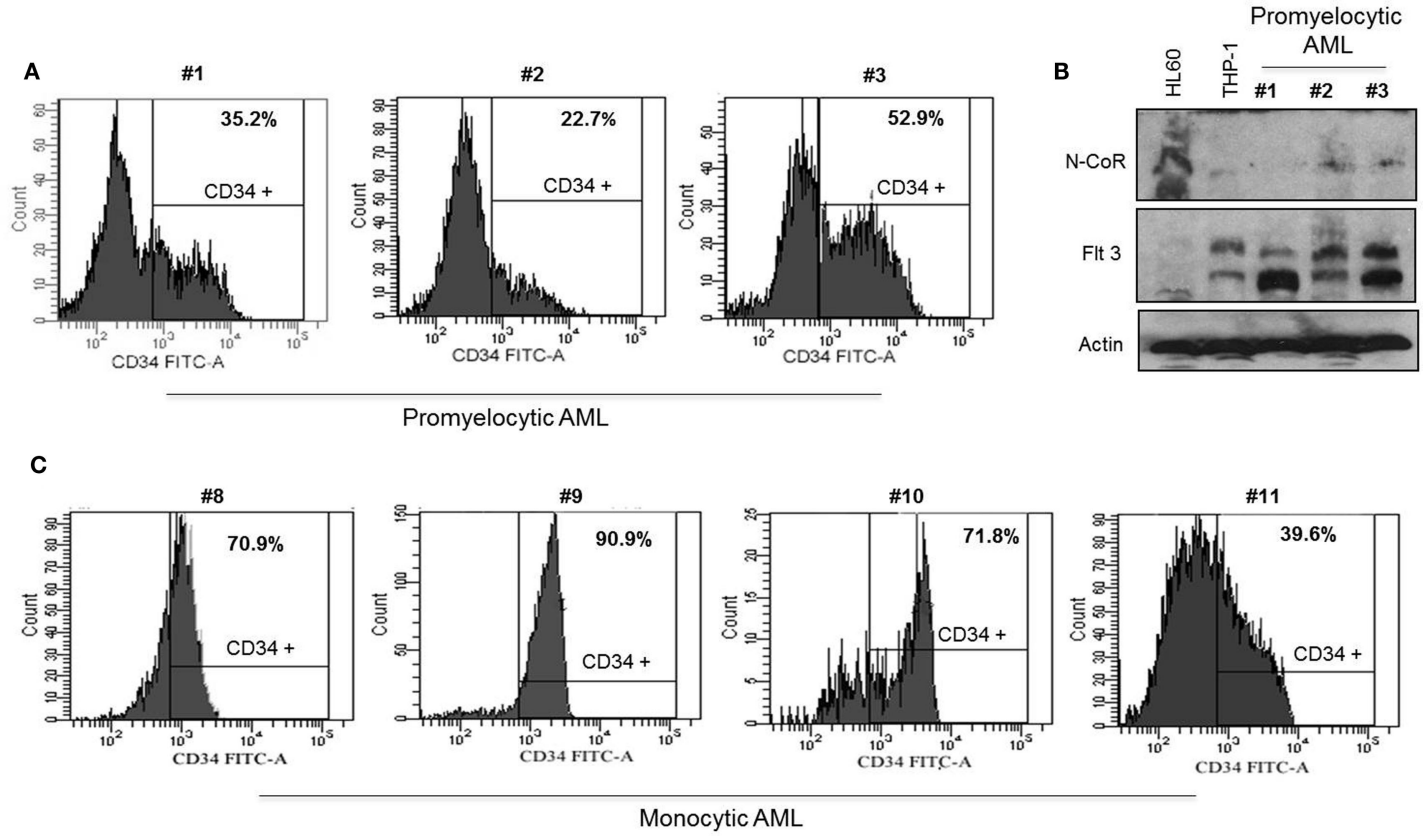
**Misfolded N-CoR is correlated to the CD34^+^/Flt3^+^-based stem cell phenotype in promyelocytic and monocytic AML primary patient samples**. **(A,C)** Percentage of CD34^+^ cells in promyelocytic AML **(A)** and monocytic AML **(C)** primary patient samples was determined by flow cytometry. **(B)** Level of N-CoR and Flt3 proteins in promyelocytic AML primary patient samples used in **(A)**. HL60 and THP-1 cells were used as positive and negative control for N-CoR protein respectively.

Our findings thus far suggested a possible role of N-CoR in the suppression of self-renewal and growth potentials of primitive hematopoietic cells, which might be necessary for their eventual commitment and maturation to cells of myeloid lineage. The suppression of this self-renewal and growth potentials by N-CoR might be a net outcome of N-CoR-mediated transcriptional repression of stem cell self-renewal genes like *Flt3*. If true, then loss of N-CoR function due to misfolding may lead to de-repression of stem cell self-renewal genes like *Flt3* and eventual reactivation of stem cell phenotypes in promyelocytic and monocytic AML. We have previously reported that primary and secondary leukemic cells derived from promyelocytic and monocytic AML harbor a distinctly misfolded and non-functional N-CoR protein while the N-CoR presented in leukemic cells derived from other AML subtypes is natively folded and functionally stable (6–9). We have further shown that a natively folded N-CoR actively represses *Flt3*, the key regulator of the stem cell phenotype and self-renewal potentials in primitive hematopoietic cells, and that misfolding of N-CoR is directly linked to up-regulation of *Flt3* in promyelocytic and monocytic AML cells (8). Besides *Flt3*, the basic stem cell phenotype in the hematopoietic system is characterized by the cell surface expression of myeloid antigen-based stem cell marker CD34. In clinical AML studies, the overall CD34 positivity of a specific AML subtypes is largely defined by the presence of at least 10–20% CD34-positive cells determined by FACS (24, 25). Therefore, to test if misfolding of N-CoR leads to the unmasking of stem cell-like phenotypes in promyelocytic and monocytic AML, the relative “stemness” (CD34^+^/Flt3^+^ phenotype) of promyelocytic and monocytic AML-derived cells was compared with that of non-promyelocytic–monocytic AML cells in which N-CoR is not misfolded. In standardized FACS assay, the CD34 positivity of promyelocytic AML-derived cells (NB4; 41.0%) and monocytic AML-derived cells [THP-1 (72.2%), Nomo-1 (18.9%), MV4-11 (40.9%), SigM5 (15.3%), and MM-1 (66.0%)] (Figure 4A) was significantly higher than minimum accepted level of 10–20% CD34 positivity for AML. By contrast, the CD34 positivity of U937 and HL-60 cells, two non-promyelocytic/monocytic AML-derived cells that harbor natively folded and fully functional N-CoR protein, was only 0.4 and 2.1%, respectively (Figure 4A). Consistent with our previous finding, all the CD34-positive promyelocytic and monocytic AML-derived cells harbored a distinctly misfolded and unstable N-CoR protein (Figure 4B) along with significantly higher level of Flt3 protein (Figure 4C). As observed with CD34 level, the level of Flt3 in non-promyelocytic/monocytic AML-derived HL-60 and U937 cells was significantly lower when compared with its level in promyelocytic and monocytic AML cells (Figure 4C). The same inverse correlation between misfolded N-CoR and Flt3/CD34 level was also observed in multiple primary promyelocytic AML patient samples (Figures 5A,B). Moreover, as observed with all the monocytic AML-derived cell lines in Figure 4A, the CD34 positivity of primary leukemic cells derived from four independent monocytic AML patients was also significantly higher (Figure 5C). All of these four primary monocytic AML patient samples, as shown previously [monocytic AML patient samples #8–#11; Figure 2 in Ref. (8)], harbored a distinctly misfolded N-CoR protein along with a significantly higher level of Flt3. Together, these findings suggest a key role of misfolded N-CoR in the up-regulation of Flt3^+^/CD34^+^-based stem cell phenotype in promyelocytic and monocytic AML cells.

### Genetic or therapeutic restoration of N-CoR conformation suppresses the CD34^+^/Flt3^+^-based stem cell phenotype in promyelocytic and monocytic AML

**Figure 6:**
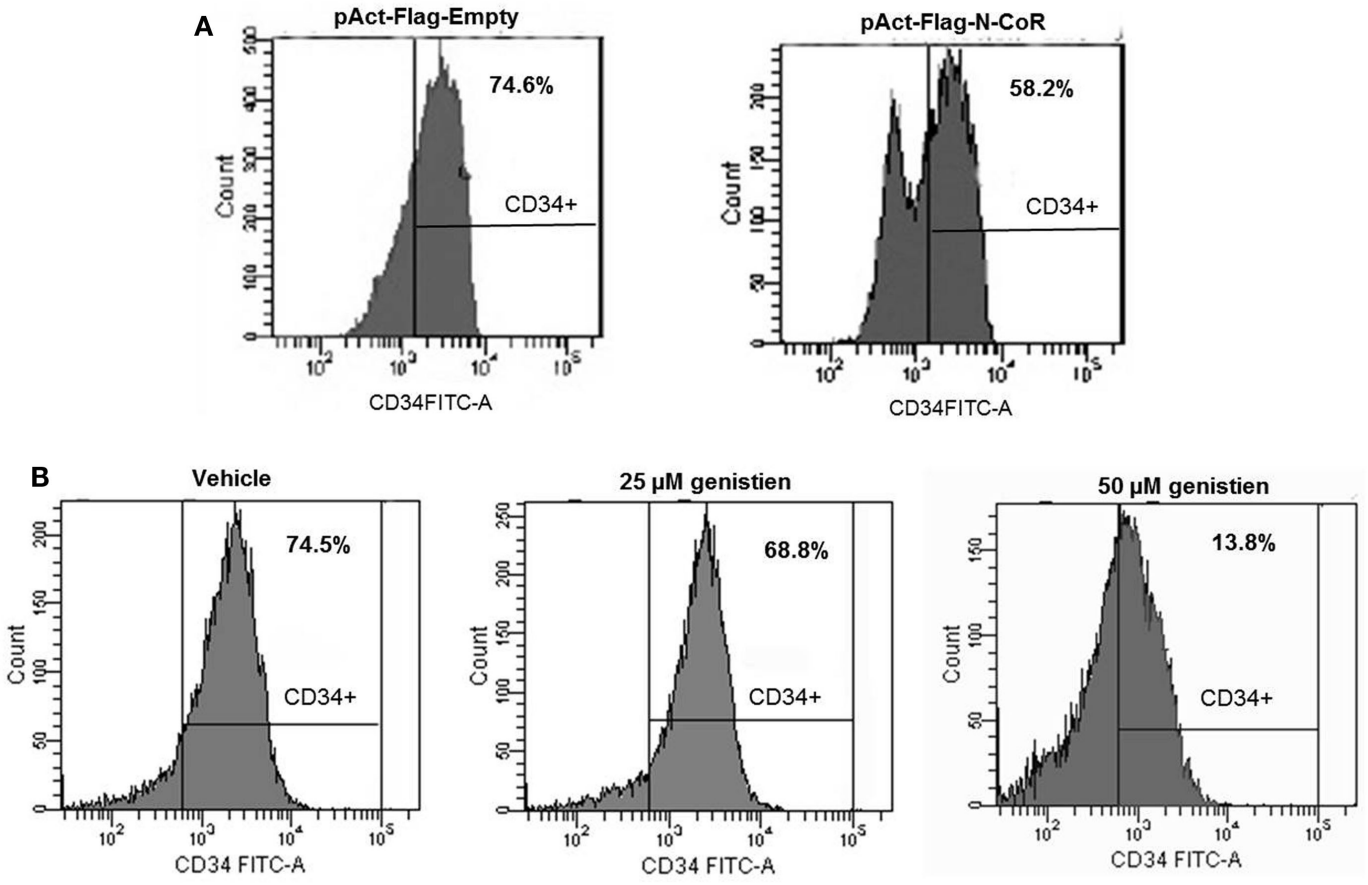
**Native N-CoR down regulates the CD34 positivity in monocytic AML cells**. **(A)** Ectopic overexpression of N-CoR in THP-1 cells reduced the CD34^+^ positivity as determined by flow cytometry. **(B)** Genistein decreased the percentage of CD34^+^ positivity of THP-1 cells in a dose-dependent manner.

Next, to investigate the role of N-CoR in the suppression of stem cell phenotype defined by Flt3^+^/CD34^+^, monocytic AML-derived THP-1 cells were transfected with Flag-tagged N-CoR expression plasmid or control vector and any change in CD34 positivity was determined by FACS. The 74.6% CD34 positivity of THP-1 cells was reduced to 58.2% after ectopic expression of N-CoR protein, suggesting that ectopic N-CoR may have contributed to a relatively moderate level of CD34 reduction in THP-1 cells (Figure 6A). This finding is in agreement with our previously published data that showed similar reduction in *Flt3* level by ectopic N-CoR in N-CoR-transfected THP-1 cells (8). To define further the role of N-CoR in the regulation of Flt3^+^/CD34^+^-based stem cell phenotype in promyelocytic and monocytic AML, we took advantage of genistein, a soy extract that effectively restored the native N-CoR conformation and function in promyelocytic and monocytic AML (7–9). As expected, genistein caused a dose-dependent reduction of CD34 positivity in THP-1 cells (Figure 6B). The reduction of CD34 positivity in THP-1 cells by genistein was most noticeable at 50 μM concentration, the same dose that most effectively stabilized the native N-CoR conformation and down-regulated the *Flt3* level in THP-1 cells (8). These findings suggest a significant correlation between N-CoR conformation and Flt3^+^/CD34^+^-based stem cell phenotype in promyelocytic and monocytic AML cells.

## Discussion

Dynamic regulation of transcription of self-renewal and lineage-specific genes by transcriptional factors and their associated co-factors play a major role in hematopoietic lineage determination, differentiation, as well as in the development of LSCs in AML. Although recent advances in the field of hematopoietic stem cell research has greatly improved our understanding about the role of specific transcription factors in the regulation of HSCs’ growth and lineage specification as well as their involvement in specific AML subtypes, very little is known about how the function of transcriptional co-factors, especially those involved in transcriptional repression, is modulated to allow selective temporal or spatial expression or suppression of various stage or lineage-specific genes in normal hematopoiesis and in AML. It is likely that these transcriptional co-factors may also play fundamental roles in the development and growth of normal as well as LSC phenotypes through their spatial and temporal regulations of self-renewal and lineage-specific genes in normal hematopoiesis and in leukemia. Based on this premise, we investigated the role of N-CoR, a key component of the generic transcriptional repressor complex essential for the transcriptional repression meditated by various transcriptional factors involved in normal hematopoiesis and in leukemia. The finding presented in this article, for the first time, identified key roles of native and misfolded N-CoR in hematopoietic stem cells development and in the formation of subtype-specific LSCs defined by myeloid antigen-based stem cell marker CD34 and the expression of key self-renewal gene *Flt3*. Based on the findings presented in this article, we can postulate that lack of expression of N-CoR in primitive hematopoietic cells may allow these cells to generate a stem cell niche by facilitating the unhindered expression of stem cell self-renewal genes like *Flt3*. However, as the primitive hematopoietic cells proceed toward maturation, the gradual increase in N-CoR expression would actively repress the Flt3, leading to suppression of cellular self-renewal potentials and down regulation of CD34 level, which will facilitate the commitment and maturation of primitive hematopoietic cells to myeloid lineage. Owing to the misfolding and premature loss of N-CoR protein in promyelocytic and monocytic AML cells, the repressive control of N-CoR on Flt3 will be lost, which would lead to up-regulation of Flt3 and eventual reactivation of the Flt3^+^/CD34^+^-based stem cell phenotype in promyelocytic and monocytic AML.

The possible role of misfolded N-CoR in the development of LSCs, as described by us in this report, might be surprising but not quite unexpected. The pathways linked to N-CoR misfolding have already been known to play important role in AML pathogenesis and has recently been identified to play a key role in the growth and development of LSC phenotypes as well. For example, Akt, which is linked to N-CoR misfolding through its kinase activity (9), has recently emerged as an important regulator of cancer stem cell growth and development. Both promyelocytic and monocytic AML are characterized by reversible differentiation blockage in which a crucial role of Akt has recently been identified (26). In a recently published study, selective Akt activation was observed in a subset of CD34^+^ cells when primary AML cells were treated with stem cell factor (27). In another study, the kinase activity of Akt was associated with the proliferation of stem cell-like cancer cells (28). A role of Akt in the enhancement of self-renewal potentials of human pluripotent stem cells in response to Wnt signaling was also recently identified (29). These findings, along with our previous report demonstrating a key role of Akt in N-CoR misfolding (9), suggest that Akt activation may act as a bridge between the extracellular oncogenic stimulus and N-CoR-based transcriptional machinery to dynamically regulate the expression of self-renewal genes during normal hematopoiesis and AML pathogenesis.

Although AML has long been recognized as a stem cell disorder, the origin of LSC in AML was always a matter of intense debate. Owing to the conflicting reports emerging from seemingly identical studies, both HSCs and committed myeloid cells were proposed as the seed of LSCs in AML. In the stem cell origin of cancer model, it was postulated that since these cells already possess unlimited self-renewal and proliferative potentials, any oncogenic alteration introduced in these cells will have better chance of transforming the cells in the long run. Another argument in favor of stem cell origin of cancer model in AML is their significantly longer life span, which would naturally allow significantly higher number of oncogenic aberrations to accumulate in the mutant clone in due course of time. The committed myeloid cells, in contrast, were considered unlikely candidates of LSC or LICs due to their limited life span and their lack of any self-renewal and proliferative potentials. However, several elegant studies in mice involving the promyelocytic and monocytic AML-specific fusion oncogenes demonstrated that these fusion oncogenes could initiate full-blown AML only when they were expressed in the committed myeloid compartment. Subsequently, it was shown that fusion oncogenes linked to promyelocytic and monocytic AML may impart or reactivate the stem cell-like unlimited self-renewal and proliferative potentials in committed cells, leading to the formation LSCs in the long run (13–17). However, the molecular mechanism underlying the reactivation of stem cell-like properties in the committed myeloid cells was largely unknown. The finding presented in this article, for the first time, identified misfolded N-CoR as the key factor linked to the ectopic reactivation of certain stem cell phenotypes in specific subtypes of AML and provided a novel mechanistic insight into the possible origin of LICs or LSCs in AML.

Although considerable progress has been made in the identification and characterization of stem cell populations in normal and malignant tissues, differentiating the LSCs from its normal counterpart for tangible diagnostic or therapeutic application is still a daunting task technically. Currently known genetic, immune-phenotypic, and functional characteristics based on which hematopoietic stem cells are characterized are also present in LSCs derived from various AML subtypes and therefore are not suitable for selective identification and targeting of LSC populations in AML. Identification and characterization of biomarkers that could differentiate the LSCs from the normal hematopoietic stem cell populations are not only important for understanding the biological process of stem cell growth but are also crucial for the design and development of highly selective and less toxic therapeutic approaches for AML. Based on the status of N-CoR protein, AML could be classified into two broader subgroups: one that originated in the stem cell compartment and have an intact N-CoR protein and the other that has its origin in relatively matured cells which harbor a misfolded and functionally redundant N-CoR protein. Identification and characterization of misfolded N-CoR protein as a molecular target and as a subtype specific biomarker of AML will facilitate better understanding of the molecular mechanisms underlying AML pathogenesis and will provide a common diagnostic and therapeutic target across all promyelocytic and monocytic AML variants.

## Experimental Procedures

### Cell lines, antibodies, and reagents

The monocytic AML cell lines [THP-1, Mono-Mac-1 (MM1), Nomo-1, and MV4-11], non-monocytic AML cell lines (U937 and HL-60), and promyelocytic AML cell line (NB4) were maintained in RPMI 1640 medium (Life Technologies, Gaithersburg, MD, USA) supplemented with 10% fetal bovine serum (FBS; Hyclone Laboratories, Logan, UT, USA), 100 U/ml penicillin, and 100 U/ml streptomycin in a humidified atmosphere of 5% CO_2_. The AML-M5 cell line SigM5 was maintained in Isocove’s modified medium (Life Technologies, Gaithersburg, MD, USA) supplemented with 20% FBS. The N-CoR (C-20) (goat polyclonal) antibody was purchased from Santa Cruz Biotechnology (CA, USA) and used as described previously (6, 7). Flt3 (C-20) antibody was purchased from Santa Cruz Biotechnology (CA, USA) while PE-conjugated anti-mouse c-kit antibody was purchased from Pharmingen (San Diego, CA, USA). N-CoR-stabilizing agent genistein was used as previously described (6, 7). Phoenix-Eco packaging cell line was a kind gift from Dr. Motomi Osato (Cancer Science Institute of Singapore, NUS, Singapore).

### Patients and primary leukemic sample preparation

Primary leukemic samples used in this study were obtained at the time of diagnosis. Diagnoses of AML were made from the morphology and cytochemistry according to the French–American–British (FAB) classification as well as immunophenotypic and cytogenetic analyses. Mononuclear cells were isolated from the peripheral blood or bone marrow samples. This study was approved by the Institutional Review Board and IACUC (institutional animal care and use committee) of National University of Singapore. For human samples used in the study, informed consent was obtained from the patients in accordance with the Declaration of Helsinki.

### Retrovirus production and infection of bone marrow cells

Mouse N-CoR cDNA was cloned into the retroviral vector MIG [MSCV/Internal ribosomal entry site (IRES)/green fluorescent protein (GFP)]. After 2 weeks of selection with diphtheria toxin (1 mg/ml) and hygromycin (0.36 U/ml), approximately 2 × 10^7^ Phoenix-Eco packaging cells were transfected with 60 μg MSCV-N-CoR using FuGENE^®^6 (Roche Diagnostics, Basel, Switzerland). Supernatants were collected 24 and 48 h after transfection and centrifuged at 12,000 rpm at 4°C overnight. The retrovirus pellet was resuspended in 0.5 ml α-minimum essential medium (α-MEM; Gibco, CA, USA). Wild-type C57BL/6 mice were injected intraperitoneally with 2.5 mg of 5-fluorouracil (5-FU; Sigma-Aldrich, MO, USA) essentially as described (30). Five days after injection, BM cells were collected from limbs of mice by flushing media through the marrow of the bones, and 3 × 10^6^ cells were cultured in 2 ml of α-MEM supplemented with 10% FBS, 1% antibiotic-antimycotic (Gibco, CA, USA), and 10 ng/ml recombinant murine interleukin-3 (IL-3), 20 ng/ml interleukin-6 (IL-6), and 10 ng/ml stem cell factor (SCF, all cytokines are from Pepro Tech EC Ltd, USA). The collected retrovirus was added to the cells at day 2 and day 3 with 50 μg RetroNectin (TAKARA, Japan), and spin infection of the cells was done at 2000 rpm at 30°C for 2 h. The cells were sorted by multiparametric flow cytometry using the FACS Vantage cell sorter (BD Biosciences, CA, USA).

### Colony-forming unit-culture assay

1 × 10^4^ GFP- and c-Kit-positive cells were cultured in 35-mm plates in 1 ml methocult M3131 methylcellulose medium (StemCell Tec., Canada) containing 1% antibiotic-antimycotic supplemented with 10 ng/ml recombinant murine IL-3, 10 ng/ml SCF, 100 ng/ml G-CSF, and 10 ng/ml EPO. To compare and quantify the colony formation (one colony consisting of more than 30 cells) capacity of N-CoR or vector-infected cells, the number of granulocyte (G), macrophage (M), granulocyte–macrophage (GM), and mixed colonies (granulocyte, erythroid, monocyte, and megakaryocyte) was scored and plotted as bar graph.

### Long-term culture-initiating cell assay

1 × 10^4^ GFP- and c-Kit-positive cells were cultured for 18 days in a six-well plate on an OP9 stromal cell layer in 2 ml αMEM medium supplemented with 10% FBS, 1% antibiotic-antimycotic, and 10 ng/ml recombinant murine IL-3, 100 ng/ml G-CSF, 10 ng/ml SCF, and 10 ng/ml EPO (Pepro Tech EC Ltd, USA). Hematopoietic cells were then harvested and subjected to Wright–Giemsa staining for morphological analysis.

### Quantitative real-time RT-PCR

Total RNA was isolated using the RNeasy Mini Kit (Qiagen GmBH, Hilden, Germany). From each sample, 2 μg of RNA was converted into cDNA by oligo(dT)_18_-primed reverse transcription using SuperScript II RT First-Strand kit (Invitrogen, Carlsbad, CA, USA) as described by the manufacturer. The cDNA was subject to semiquantitative PCR analysis using Accuprime Taq polymerase system (Invitrogen, Carlsbad, CA, USA) according to manufacturer’s recommendations. Flt3 primers were used as previously described in Ref. (8).

### Detection of CD34 cell surface antigen

Cell cultures and patient samples were collected, washed twice with PBS + 0.5% bovine serum albumin, and incubated for 10 min on ice in 1 ml of PBS + 0.5% bovine serum albumin with 2 μg/ml propidium iodide (Pharmingen, San Diego, CA, USA) and FITC-conjugated monoclonal mouse anti-human CD34 antibody (1:100 dilution) or control IgG (1:100 dilution) (Miltenyi Biotech, Auburn, CA, USA). Antibody-conjugated cells were washed with PBS + 0.5% bovine serum albumin and analyzed using FACS.

## Conflict of Interest Statement

The authors declare that the research was conducted in the absence of any commercial or financial relationships that could be construed as a potential conflict of interest.

## References

[1] Rosenbauer F Tenen DG. Transcription factors in myeloid development: balancing differentiation with transformation. Nat Rev Immunol (2007) 7:105–17. 10.1038/nri2024 17259967

[2] Hörlein AJ Näär AM Heinzel T Torchia J Gloss B Kurokawa R Ligand-independent repression by the thyroid hormone receptor mediated by a nuclear receptor co-repressor. Nature (1995) 377:397–404. 10.1038/377397a0 7566114

[3] Heinzel T Lavinsky RM Mullen TM Söderstrom M Laherty CD Torchia J A complex containing N-CoR, mSin3A, and histone deacetylase mediates transcriptional repression. Nature (1997) 387:43–8. 10.1038/387043a0 9139820

[4] Nomura T Khan MM Kual SC Wadha R Colmenares C Ishii S. Ski is a component of the histone deacetylase complex required for transcriptional repression by Mad and thyroid hormone receptor. Genes Dev (1999) 13:412–23. 10.1101/gad.13.4.412 10049357 PMC316468

[5] Khan MM Nomura T Chiba T Tanaka K Yoshida H Mori K The fusion onco-protein PML-RAR induces endoplasmic reticulum associated degradation of N-CoR and ER stress. J Biol Chem (2004) 279:11814–24. 10.1074/jbc.M312121200 14701861

[6] Ng AP Nin DS Howe FJ Chen C Khan MM. Cleavage of mis-folded nuclear receptor co-repressor confers resistance to unfolded protein response-induced apoptosis. Cancer Res (2006) 66:9903–12. 10.1158/0008-5472.CAN-06-0002 17047052

[7] Ng AP Nin DS Howe FJ Venkataraman D Chen C Khan MM. Therapeutic targeting of nuclear receptor co-repressor (N-CoR) misfolding in acute promyelocytic leukemia (APL) cells with genistein. Mol Cancer Ther (2007) 6:2240–8. 10.1158/1535-7163.MCT-06-0705 17699721

[8] Nin DS Kok WK Li F Takahashi S Chng WJ Khan M. Role of misfolded N-CoR mediated transcriptional deregulation of Flt3 in acute monocytic leukemia (AML)-M5 subtype. PLoS One (2012) 7:e34501. 10.1371/journal.pone.0034501 22514634 PMC3326026

[9] Nin DS Ali A Okumura K Asou N Chen CS Chng WJ Akt-induced phosphorylation of N-CoR at serine 1450 contributes to its misfolded conformational dependent loss (MCDL) in acute myeloid leukemia of the M5 subtype. PLoS One (2013) 8(8):e70891. 10.1371/journal.pone.0070891 23940660 PMC3733915

[10] Moore MAS. Converging pathways in leukemogenesis and stem cell self-renewal. Exp Hematol (2005) 33:719–37. 10.1016/j.exphem.2005.04.011 15963848

[11] Parcells BW Ikeda AK Simms-Waldrip T Moore TB Sakamoto KM. FMS-like tyrosine kinase 3 in normal hematopoiesis and acute myeloid leukemia. Stem Cells (2006) 24:1174–84. 10.1634/stemcells.2005-0519 16410383

[12] Oancea C Rüster B Brill B Roos J Heinssmann M Bug G STAT activation status differentiates leukemogenic from non-leukemogenic stem cells in AML and is suppressed by arsenic in t(6;9)-positive AML. Gen Cancer (2014) 11(12):378–92. 10.18632/genesandcancer.39 PMC4279436 25568664

[13] Wojiski S Guibal FC Kindler T Lee BH Jesneck JL Fabian A PML–RARa initiates leukemia by conferring properties of self-renewal to committed promyelocytic progenitors. Leukemia (2009) 23(8):1462–71. 10.1038/leu.2009.63 19322209 PMC2914549

[14] Westervelt P Lane AA Pollock JL Oldfather K Holt MS Zimonjic DB A high penetrance mouse model of acute promyelocytic leukemia with very low levels of PML-RAR expression. Blood (2003) 102:1857–65. 10.1182/blood-2002-12-3779 12750176

[15] Westervelt P Ley TJ Seed vs soil: the importance of the target cell for transgenic models of human leukemias. Blood (1999) 93:2143–8. 10090920

[16] Krivtsov AV Twomey D Feng Z Stubbs MC Wang Y Faber J Transformation from committed progenitor cell to leukemia stem cells initiated by MLL-AF9. Nature (2006) 442(7104):818–22. 16862118 10.1038/nature04980

[17] Somervaille TC Cleary ML. Identification and characterization of leukemia stem cells in murine MLL-AF9 acute myeloid leukemia. Cancer Cell (2006) 10:257–68. 10.1016/j.ccr.2006.08.020 17045204

[18] Bonnet D Dick JE. Human acute myeloid leukemia is organized as a hierarchy that originates from a primitive hematopoietic cell. Nat Med (1997) 3:730–7. 10.1038/nm0797-730 9212098

[19] Lapidot T Kollet O. The essential roles of the chemokine SDF-1 and its receptor CXCR4 in human stem cell homing and repopulation of transplanted immune-deficient NOD/SCID and NOD/SCID/B2m (null) mice. Leukemia (2002) 16:1992–2003. 10.1038/sj.leu.2402684 12357350

[20] Schuringa JJ Schepers H. Ex vivo assays to study self-renewal and long-term expansion of genetically modified primary human acute myeloid leukemia stem cells. Methods Mol Biol (2009) 538:287–300. 10.1007/978-1-59745-418-6_14 19277587

[21] van Gosliga D Schepers H Rizo A van der Kolk D Vellenga E Schuringa JJ. Establishing long-term cultures with self-renewing acute myeloid leukemia stem/progenitor cells. Exp Hematol (2007) 35:1538–49. 10.1016/j.exphem.2007.07.001 17889721

[22] Warner JK Wang JC Takenaka K Doulatov S McKenzie JL Harrington L Direct evidence for cooperating genetic events in the leukemic transformation of normal human hematopoietic cells. Leukemia (2005) 19:1794–805. 10.1038/sj.leu.2403917 16094415

[23] Woehrer S Miller CL Eaves CJ. Long-term culture-initiating cell assay for mouse cells. Methods Mol Biol (2013) 946:257–66. 10.1007/978-1-62703-128-8_16 23179837

[24] Casasnovas RO Slimane FK Garand R Faure GC Campos L Deneys V Immunological classification of acute myeloblastic leukemias: relevance to patient outcome. Leukemia (2003) 17:515–27. 10.1038/sj.leu.2402821 12646939

[25] Kanda Y Hamaki T Yamamoto R Chizuka A Suguro M Matsuyama T The clinical significance of CD34 expression in response to therapy of patients with acute myeloid leukemia: an overview of 2483 patients from 22 studies. Cancer (2000) 88:2529–33. 10.1002/1097-0142(20000601)88:11<2529::AID-CNCR14>3.3.CO;2-J 10861429

[26] Sykes SM Lane SW Bullinger L Kalaitzidis D Yusuf R Saez B AKT/FOXO signaling enforces reversible differentiation blockade in myeloid leukemias. Cell (2011) 146(5):697–708. 10.1016/j.cell.2011.07.032 21884932 PMC3826540

[27] Han L Qiu P Zeng Z Jorgensen JL Mak DH Burks JK Single-cell mass cytometry reveals intracellular survival/proliferative signalling in FLT3-ITD-mutated AML stem/progenitor cells. Cytometry A (2015) 87(4):346–56. 10.1002/cyto.a.22628 25598437 PMC4388314

[28] Zhao QW Zhou YW Li WX Kang B Zhang XQ Yang Y Akt-mediated phosphorylation of Oct4 is associated with the proliferation of stem-like cancer cells. Oncol Rep (2015) 33(4):1621–9. 10.3892/or.2015.3752 25625591 PMC4358081

[29] Huang TS Li L Moalim-Nour L Jia D Bai J Yao Z A regulatory network involving β-catenin, E-cadherin, PI3K/Akt, and slug balances self-renewal and differentiation of human pluripotent stem cells in response to Wnt signalling. Stem Cells (2015) 33:1419–33. 10.1002/stem.1944 25538040 PMC5297972

[30] Motoda L Osato M Yamashita N Jacob B Chen LQ Yanagida M Runx1 protects hematopoietic stem/progenitor cells from oncogenic insult. Stem Cells (2007) 25:2976–86. 10.1634/stemcells.2007-0061 17823240

